# Systematic review, including meta-analyses, on the management of locally advanced pancreatic cancer using radiation/combined modality therapy

**DOI:** 10.1038/sj.bjc.6603719

**Published:** 2007-04-03

**Authors:** A Sultana, C Tudur Smith, D Cunningham, N Starling, D Tait, J P Neoptolemos, P Ghaneh

**Affiliations:** 1 Division of Surgery and Oncology, School of Cancer Studies, University of Liverpool, 5th Floor-UCD Building, Daulby Street, Liverpool L69 3GA, UK; 2 Centre for Medical Statistics and Health Evaluation, University of Liverpool, Shelley's Cottage, Brownlow Street, Liverpool L69 3GS, UK; 3 Department of Medicine, Royal Marsden Hospital, Downs Road, Sutton, Surrey SM2 5PT, UK; 4 Department of Clinical Oncology, Royal Marsden Hospital, Downs Road, Sutton, Surrey SM2 5PT, UK

**Keywords:** pancreas cancer, radiotherapy, chemoradiation, chemotherapy, combined modality

## Abstract

There is no consensus on the management of locally advanced pancreatic cancer, with either chemotherapy or combined modality approaches being employed (Maheshwari and Moser, 2005). No published meta-analysis (Fung *et al*, 2003; Banu *et al*, 2005; Liang, 2005; Bria *et al*, 2006; Milella *et al*, 2006) has included randomised controlled trials employing radiation therapy. The aim of this systematic review was to compare the following: (i) chemoradiation followed by chemotherapy (combined modality therapy) *vs* best supportive care (ii) radiotherapy *vs* chemoradiation (iii) radiotherapy *vs* combined modality therapy (iv) chemotherapy *vs* combined modality therapy (v) 5FU-based combined modality treatment *vs* another-agent-based combined modality therapy. Relevant randomised controlled trials were identified by searching databases, trial registers and conference proceedings. The primary end point was overall survival and secondary end points were progression-free survival/time-to-progression, response rate and adverse events. Survival data were summarised using hazard ratio (HR) and response-rate/adverse-event data with relative risk. Eleven trials involving 794 patients met the inclusion criteria. Length of survival with chemoradiation was increased compared with radiotherapy alone (two trials, 168 patients, HR 0.69; 95% confidence interval (CI) 0.51–0.94), but chemoradiation followed by chemotherapy did not lead to a survival advantage over chemotherapy alone (two trials, 134 patients, HR 0.79; CI 0.32–1.95). Meta-analyses could not be performed for the other comparisons. A survival benefit was demonstrated for chemoradiation over radiotherapy alone. Chemoradiation followed by chemotherapy did not demonstrate any survival advantage over chemotherapy alone, but important clinical differences cannot be ruled out due to the wide CI.

Pancreatic cancer is a difficult condition to treat, evidenced by the fact that the annual mortality figures are close to the incidence rate (Jemal *et al*, 2006). Ninety per cent of patients have unresectable disease at diagnosis, of whom 40–50% have locally advanced disease (White *et al*, 1999), and reported to have better median survival of 6–10 months compared to the 3–6 months noted in metastatic disease (American Cancer Society, 2003). Radiotherapy approaches, with or without chemotherapy, have been frequently used in this subset (Maheshwari and Moser, 2005).

Previous meta-analyses in this area have looked at chemotherapy and novel agents (Fung *et al*, 2003; Banu *et al*, 2005; Liang, 2005; Bria *et al*, 2006; Milella *et al*, 2006), and the Cochrane Collaboration (Yip *et al*, 2006) have done a recent systematic review on advanced pancreatic cancer that included a qualitative overview of trials involving radiotherapy, but there has been no meta-analyses performed to date addressing this treatment option. Evaluating this approach is important, as currently there is no uniformly agreed standard of care in the management of patients with locally advanced disease.

We have attempted an up-to-date analysis of the different radiotherapeutic options employed in locally advanced pancreatic cancer, thereby including an area not covered by previous meta-analyses. Furthermore, we have adopted the most appropriate statistical methods for meta-analysis of time to event data extracted from published reports (Parmar *et al*, 1998).

## METHODS

### Aims

To review systematically the published and unpublished literature, comparing the following therapies:

1. Chemoradiotherapy, followed by chemotherapy *vs* best supportive care
2. Radiotherapy *vs* chemoradiotherapy
3. Radiotherapy *vs* chemoradiotherapy, followed by chemotherapy
4. Chemotherapy *vs* chemoradiotherapy, followed by chemotherapy (combined modality therapy)
5. 5FU-based chemoradiotherapy followed by chemotherapy *vs* another agent-based chemoradiotherapy, followed by chemotherapy

### Search strategy

Trials were identified by searching MEDLINE, OLDMEDLINE (1950–1965), EMBASE (1974 to date), ISI Web of Science (incorporating Science Citation Index 1945 to date; ISI Science and Technology Proceedings 1990 to date; CancerLit (1960 to date) and Current contents databases (from 1996 to date) as far back as they go. In addition, trial registries (Registries of the National Cancer Institute Physician Data Query, the UK Co-ordinating Committee on Cancer Research, National Clinical Trials Registry, and the Cochrane Controlled Trials Register) and conference proceedings (American Society of Clinical Oncology, American Association of Cancer Research and the European Cancer Conference, European Society of Medical Oncology, American Gastroenterological Association, European Pancreatic Club, American Association of Pancreatology, British Society of Gastroenterology, and the United European Gastroenterology Week) were searched. References of selected papers and previous systematic reviews were scanned for any other relevant trials, and original trialists were contacted for possible unpublished trials.

### Selection criteria

Randomised controlled trials were selected based on their abstract, or if that was unclear, the paper. Inclusion criteria were randomised controlled trials involving patients with advanced pancreatic cancer of the exocrine pancreas, comparing the therapies listed above. The exclusion criteria were trials which were nonrandomised, or included surgical resection of tumours and cancers other than pancreas cancers, wherein data was not available for the pancreas cancer subset. The study selection was done by two independent assessors, with any difference of opinion sorted by discussion.

### End points

Overall survival (OS) defined as time from randomisation to death, was the primary outcome measure. Alternative definitions such as time from initiation of therapy to death were also included and noted as a potential source of heterogeneity.

Progression free survival (PFS) or time to progression (TTP), overall response rate (ORR) and adverse events (AE) were the secondary outcome measures. PFS was defined as time from randomisation to progression or death. Time to progression (TTP) was defined as the time from randomisation to disease progression. PFS was analysed separately from TTP as the former accounts for all deaths as well as progression events, whereas the latter only accounts for progression events. Overall response rate (ORR) was defined as the number of partial and complete responses and adverse events defined as side effects occurring from the date of randomisation till either end of study or death.

### Quality assessment

Methodological quality was assessed based on the method of allocation generation (method of randomisation), allocation concealment (where the randomisation was carried out), blinding and losses to follow up. These were classified as adequate, inadequate or unknown, and the results of the different components discussed qualitatively.

### Data extraction

Data extraction was performed independently by two reviewers using a standardised data extraction sheet. Disagreements were resolved by discussion and any data uncertainties forwarded to the original trialist for clarification.

### Statistical analysis

Individual trial level time to event data (OS and PFS/TTP) were summarised by the log hazard ratio (HR) and its variance. As many trials do not report this information directly (Altman *et al*, 1995), appropriate data such as log rank test results were extracted to allow estimation of the log HR and its variance using previously reported methods (Parmar *et al*, 1998; Williamson *et al*, 2002). One of these approaches relies on extracting data from published survival curves (Parmar *et al*, 1998). The software we used (version 3.0; 28 September, 2004) to estimate the trial level log HR and variance-based on summary data extracted from published survival curves was developed by Matthew Sydes and Jayne Teirney of the MRC Clinical Trials Unit, London. Trial-level log HRs and their variances were entered into RevMan version 4.2 (a Windows-based software package used by the Cochrane Collaboration for writing systematic reviews and undertaking meta-analysis Sterne *et al*, 2001) and pooled using an inverse variance weighted average with results presented as a HR and 95% confidence interval (CI).

Dichotomous data (response rate/adverse events) were summarised using relative risks and 95% CIs with the Mantel–Haensel method used for pooling results across trials (Deeks *et al*, 2001).

Heterogeneity was assessed by visual inspection of the forrest plot, the Cochran's *χ*^2^ test (using a 10% significance level) and interpretation of the *I*^2^ statistic (percentage of variation due to heterogeneity with higher values indicating a greater degree of heterogeneity) (Deeks *et al*, 2004). The factors set out *a priori* to investigate heterogeneity were age, gender, performance status, previous treatment, site of the cancer (head, body or tail), and the chemotherapy/radiation used with the dose, combinations, and frequency. A fixed effect (FE) approach was adopted unless there was evidence of significant heterogeneity that could not be adequately explained, in which case a random effects (RE) approach was used.

Publication bias was assessed by visual inspection of funnel plots (Light and Pillemer, 1984).

## RESULTS

Eleven trials involving 794 patients met the reviews' inclusion criteria and six of these trials involving 451 patients were included in the meta-analyses. The quality of included studies is described in Table 1.

As there was only one study identified in two comparisons viz., chemoradiation, followed by chemotherapy *vs* best supportive care (BSC) comparison (Table 2) (Shinchi *et al*, 2002), and radiotherapy *vs* chemoradiation, followed by chemotherapy comparison (Table 3) (Moertel *et al*, 1981), a meta-analysis could not be undertaken for these comparisons.

In the single randomised controlled trial examined (Shinchi *et al*, 2002), there was survival advantage for chemoradiation followed by chemotherapy, over BSC (1 trial, 31 patients, HR 0.28; 95% CI 0.13–0.60). The median time-to-progression was 6.1 months, with overall response rate of 31% (5 out of 16) in the treated group but corresponding data were not provided for the BSC group. A quarter of treated patients (4 out of 16) developed complications secondary to the chemoradiation, with nausea occurring in three patients and one experiencing grade 2 leucopenia.

There was survival advantage with chemoradiotherapy followed by chemotherapy over radiation alone (1 trial, 56 patients, HR 0.50; 95% CI 0.29–0.84) in the trial conducted by Moertel *et al* (1981). Time to progression was also better in the chemoradiotherapy followed by chemotherapy arm over radiotherapy alone arm (HR 0.51; 95% CI 0.32–0.81).

Three trials (256 patients) met the inclusion criteria for the comparison of 5FU-based multimodality therapy *vs* another-agent-based multimodality therapy (Table 4). However, meta-analyses were not performed, as the studies were too clinically heterogeneous to be grouped together in a clinically meaningful analysis. The agents used for radio sensitisation in the non-5FU arm were different in all three trials, with gemcitabine alone (Li *et al*, 2003), gemcitabine+cisplatin (Wilkowski *et al*, 2006) and adriamycin (Gastrointestinal Tumour Study Group, 1985) being used.

Adriamycin-based multimodality therapy, using split course radiotherapy given via two portals, did not demonstrate a significant survival advantage over 5FU-based treatment (HR for 5FU *vs* Adriamycin=0.97 95% CI 0.73–1.29), accompanied by the drawback of significantly increased adverse events (*P*<0.05) (Gastrointestinal Tumour Study Group, 1985).

A randomised controlled trial of 34 patients found significantly improved overall survival (14.5 *vs* 6.7 months), time to progression (7.1 *vs* 2.7 months) and response rate (50 *vs* 13%) in patients treated with gemcitabine-based chemoradiation (600 mg m^−2^week^−1^ for 6 weeks), followed by gemcitabine, in comparison to a control arm of 5FU-based chemoradiation (500 mg m^−2^ day^−1^ for 3 days repeated every 2 weeks for 6 weeks), followed by gemcitabine (Li *et al*, 2003). Toxicity between the two arms was similar and radiation had been given using three-dimensional conformal radiotherapy. These results were not borne out in a recent randomised controlled trial of 65 patients (preliminary results), which did not find improvement in 9 month survival for a group treated with gemcitabine and cisplatin chemoradiation, *vs* another treated with protracted venous 5-FU infusion chemoradiation (Wilkowski *et al*, 2006).

### Comparison of radiotherapy *vs* chemoradiotherapy

Two randomised controlled trials with 168 patients were included in this analysis (Table 5) (Moertel *et al*, 1969; Cohen *et al*, 2005). One study described adequate methods of allocation generation, one described adequate methods of concealment, and both described adequate losses to follow-up. One trial was blinded (Table 1).

**Figure 1:**
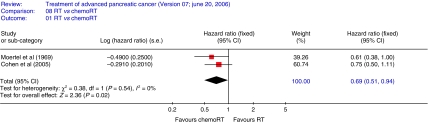
Overall survival-radiotherapy *vs* chemoradiotherapy. The plot demonstrates a 31% reduction in risk of death following chemoradiotherapy, compared to radiation alone (two trials 168 patients HR 0.69; CI 0.51–0.94 (FE)).

The HR summarises survival for chemoradiotherapy compared to radiotherapy with HR<1 indicating a survival advantage for chemoradiotherapy. Overall survival (Figure 1) was significantly better, with a 31% reduction in risk of death following chemoradiotherapy, compared to radiation alone (two trials 168 patients HR 0.69; CI 0.51–0.94 (FE)).

**Figure 2:**
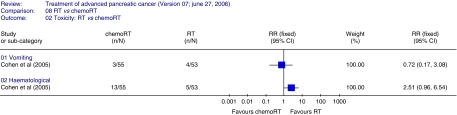
Adverse events radiotherapy *vs* chemoradiotherapy. The plot demonstrates vomiting and haematological toxicity adverse events, haematological toxicity was lower in the radiotherapy arm compared to the chemoradiation arm.

AE data could only be assessed for two parameters, vomiting and haematological toxicity, and the latter was lower in the radiotherapy arm compared to the chemoradiation arm (Figure 2). Cohen *et al* (2005) did not find any difference in disease-free survival (HR 0.77; 95% CI 0.52–1.14) or response rate between the two treatments (RR 1.48; 95% CI 0.37–5.89).

### Comparison of chemotherapy to chemoradiotherapy, followed by chemotherapy

Four randomised controlled trials (Table 6) with 283 patients were included (Hazel *et al*, 1981; Klassen *et al*, 1985; Gastrointestinal Tumour Study Group, 1988; Chauffert *et al*, 2006), but overall survival data for time-to-event analysis was only available in two studies (134 patients) (Hazel *et al*, 1981; Klassen *et al*, 1985). Adequate methods of allocation generation were described in two studies, adequate methods of concealment in one study and adequate losses to follow-up in 3. No study was blinded (Table 1).

**Figure 3:**
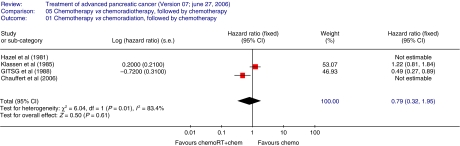
Overall survival-chemotherapy *vs* chemoradiotherapy, followed by chemotherapy. The plot demonstrates that overall survival was not significantly better in the chemoradiation followed by chemotherapy arm compared to the chemotherapy only arm (two trials 134 patients HR 0.79; 95% CI 0.32–1.95 (RE)) There was significant heterogeneity between the two trials analysed (*P*=0.01; *I*^2^=83.4%).

The HR summarises survival for chemoradiotherapy, followed by chemotherapy compared to chemotherapy with HR<1 indicating a survival advantage for chemoradiotherapy, followed by chemotherapy. Overall survival (Figure 3) was not significantly better in the chemoradiation, followed by chemotherapy arm compared to the chemotherapy only arm (two trials 134 patients HR 0.79; 95% CI 0.32–1.95 (RE)) but the wide CI includes clinically significant differences in both directions). There was significant heterogeneity between the two trials analysed (*P*=0.01; *I*^2^=83.4%).

The Klassen study found no significant difference in time to progression between the two arms (HR 1.03; 95% CI 0.73–1.47). No other end points could be analysed for this comparison, owing to inadequate published data.

#### Publication bias

Despite our exhaustive searches, examination of the funnel plots revealed evidence of bias, possibly publication bias, for all comparisons assessed. However, due to the small number of trials included within most comparisons, interpretation of funnel plots is difficult.

## DISCUSSION

This systematic review includes 11 studies that randomised 794 patients with locally advanced pancreas cancer and represents the only meta-analyses to date that examine the use of radiotherapeutic approaches in locally advanced pancreas cancer. Compared with the Cochrane Collaboration review (Yip *et al*, 2006), our review excluded two of their studies (Childs *et al*, 1965; Earle *et al*, 1994) but included three additional recent randomised controlled trials (Cohen *et al*, 2005; Chauffert *et al*, 2006; Wilkowski *et al*, 2006). The study conducted by Childs *et al* (1965) was reported in final form by Moertel *et al* (1969), and hence the exclusion of the duplicate former study, whereas the study by Earle *et al* (1994) did not fit into the comparisons that were being assessed. The most appropriate statistical methods for meta-analysis of time to event data extracted from published reports have been used in our report (Parmar *et al*, 1998).

We did not find any randomised controlled trials that compared radiation alone or chemoradiation alone to BSC. The basis for incorporating radiation therapy in pancreatic cancer was based on a Mayo clinic randomised controlled trial that randomised patients to receive radiotherapy or 5FU-based radiotherapy (Moertel *et al*, 1969) and an uncontrolled study of 23 patients who received radiotherapy (5040–6680 rad), with 13 patients also receiving 5FU (Haslam *et al*, 1973). Median survival in the study by Haslam *et al* (1973) was 7.5 months.

One small randomised controlled trial (Shinchi *et al*, 2002) of 31 patients compared chemoradiation, followed by chemotherapy, to BSC. 5FU (200 mg m^−2^ day^−1^) was administered for the duration of the radiation therapy, followed by 500 mg m^−2^per week thereafter, until progressive disease or unacceptable toxicity occurred. The regimen of daily 5FU concomitant with radiation differs from all the other randomised controlled trials using chemoradiation (Moertel *et al*, 1969; Hazel *et al*, 1981; Klassen *et al*, 1985; Gastrointestinal Tumour Study Group, 1988; Cohen *et al*, 2005; Chauffert *et al*, 2006), wherein weekly 5FU (500–1000 mg m^−2^ given either weekly or on first and last 3 days of radiotherapy) was used for radio sensitisation. A nonsignificant reduction in liver and peritoneal metastases was seen in the treatment arm. This finding, along with significant improvement in overall survival, may well be an effect of the chemotherapy rather than the radiation. The fact that the majority of patients died of local disease progression (62%) in the treatment arm supports this possibility.

Overall survival was better with chemoradiation compared to radiotherapy alone. Although there was no statistical heterogeneity between these two trials, both the inclusion criteria and radiation techniques differed. Moertel *et al* (1969) staged patients using clinical and surgical techniques, whereas Cohen used extensive imaging, in the form of CT scan of abdomen, chest X-ray and bone and brain scan, followed by surgical staging. Thus, selection criteria were more stringent in the latter study. Radiation techniques have also improved between the 1960s, when Moertel published his findings, to the 1980s, when the Cohen study was open to accrual. The latter study questions the merit of combining these two modalities, in the light of low response rate, poor survival and increased toxicity. Moreover, neither radiotherapy nor chemoradiation address the micro metastases present in patients labelled as locally advanced cancer (Liu and Traverso, 2004; Shoup *et al*, 2004).

The 1981 GITSG study was instrumental in popularising multimodality therapy in the treatment of locally advanced pancreas cancer, as it showed a doubling of survival duration over radiation alone (Moertel *et al*, 1981). This was at the price of greater toxicity, as myelosuppression was more frequent and severe, and two cases of gastrointestinal bleeding and one instance of moderate azotemia were reported in the combined modality arm.

Meta-analysis of chemotherapy *vs* chemoradiation, followed by chemotherapy in the two evaluable trials, found no significant difference between the two approaches, in the presence of inter-study heterogeneity. This could be owing to the following factors:

Difference in radiation. The GITSG study (Gastrointestinal Tumour Study Group, 1988) utilised 54 Gy, given via three or four fields whereas the Klassen study (Klassen *et al*, 1985) used 4000 rad given by parallel-opposed anterior and posterior portals.
The chemotherapy agents also differed, with the GITSG study using a combination of 5FU, streptozotocin and mitomycin C (SMF), whereas the Klassen study used single agent 5FU.

The difference in effects between the two studies could be due to the difference in the radiotherapy used, as the GITSG study concluded that the SMF regimen did not prove to be superior to single agent 5FU. The CIs here are very wide, with reduction in risk of death with chemoradiation followed by chemotherapy being, on one end of the spectrum, as much as 68%, whereas at the other end, the increase in risk of death being 95% greater compared to chemotherapy alone. Another point to be borne in mind was that the GITSG study had closed prematurely, owing to lack of funding, with the total number of patients accrued only a third of the planned sample size. Early stoppage of a trial could lead to an erroneous estimation of treatment effects, with a propensity for exaggeration, that is, a random high (Schulz and Grimes, 2005).

For the two studies (Hazel *et al*, 1981; Chauffert *et al*, 2006) in this comparison wherein we were unable to calculate HR from the published data, the overall results did not support the use of chemoradiotherapy followed by chemotherapy, over chemotherapy alone. In the trials conducted by Hazel *et al* (1981) of 30 patients, there was no significant difference in median survival between the two arms (7.3 months in multimodality treatment arm *vs* 7.8 months in the chemotherapy arm). A recent randomised controlled trial, done nearly two decades after the GITSG study, found significant survival advantage (log rank *P*=0.014) with gemcitabine single agent chemotherapy (median survival 14.3 months) over multimodality therapy in locally advanced disease (median survival=8.4 months), necessitating early stoppage of the trial (Chauffert *et al*, 2006). All studies found greater haematological toxicity in the multimodality treatment arm, and the Chauffert study found a higher incidence of nonhaematological toxicity as well.

To conclude, survival benefit was demonstrated for the comparison of chemoradiotherapy over radiation alone, with evidence from a single randomised controlled trial demonstrating survival benefit for chemoradiotherapy followed by chemotherapy over radiation alone and chemoradiotherapy followed by chemotherapy over BSC. There is insufficient evidence to support the use of chemoradiation with follow on chemotherapy over chemotherapy alone in the absence of a survival advantage, coupled with greater toxicity. However, the wide CIs make it difficult to rule out important clinical differences. The results of the Intergroup study E4201, which aimed to compare gemcitabine alone to gemcitabine and radiation therapy would have helped settle the issue of whether there is a role, if at all, for multimodality therapy in locally advanced pancreatic cancer (Lockhart *et al*, 2005). Unfortunately, this trial was closed owing to poor accrual and hence the question remains unanswered (Cardenes *et al*, 2006).

There are missing links in the chain of evidence using radiation therapy in advanced pancreas cancer, in particular, the fact that at inception, radiation alone was not compared against BSC in a randomised setting, unlike with chemotherapy approaches. In addition, there are several small inadequately powered randomised controlled trials testing different hypothesis, with a missing golden thread in the evolution of these studies. Staging has improved over time, with significant advances in imaging in the last 5 years following the advent of multidetector row helical CT with or without positron emission tomography (Michl *et al*, 2005). The frontline approaches to staging today are contrast-enhanced multi-detector row helical CT, with its high sensitivity for identifying vascular invasion, and endoscopic ultrasound, which can pick up tumours as small as 2–3 mm. In the event of these modalities being equivocal, there are additional tools available in the form of MRI with MR-angiography, MRCP, PET/CT and staging laparoscopy. Radiotherapy has also evolved, from the two-dimensional split course radiation encompassing larger treatment volumes with resultant toxicity, to the newer, more targeted three-dimensional conformal radiation and the intensity modulated radiation therapy (IMRT) approaches (Garofalo *et al*, 2006). Image-guided radiation therapy (IGRT) takes into account the interfraction and intrafraction dose variation, as a consequence of organ motion. Better technology and the use of conformal treatment have led to higher tolerable radiation doses (Yang *et al*, 2005). With improvements in staging and radiation techniques future studies may re-evaluate the application of upfront chemoradiation or the use of early systemic therapy for the treatment of micro metastases followed by consolidation therapy within adequately powered studies.

To conclude, we advocate the use of chemotherapy in patients with locally advanced cancer, as currently there is insufficient evidence to endorse the use of chemoradiation, followed by chemotherapy, over chemotherapy alone. This recommendation is also supported by a recent meta-analysis, which demonstrated a significant survival benefit for chemotherapy over BSC and gemcitabine-based combinations over single agent gemcitabine in patients with advanced pancreatic cancer (Sultana *et al*, 2007). It is important to bear in mind that no randomised controlled trial has compared radiotherapy to chemotherapy and the single randomised controlled trials that compared chemoradiation, followed by chemotherapy to either BSC or radiation, are small. With improvements in staging and radiation techniques future trials may influence these recommendations.

## Figures and Tables

**Table 1:** Quality of included studies

**Comparison**	**Trial**	**Allocation sequence generation**	**Allocation concealement**	**Blinding**	**Follow-up**
Chemoradiotherapy followed by chemotherapy *vs* BSC	Shinchi *et al* (2002)	Unclear	Unclear	Not performed	Adequate
Radiotherapy *vs* chemoradiotherapy, followed by chemotherapy	Moertel *et al* (1981)	Adequate	Adequate	Not performed	Adequate
5FU-based chemoradiotherapy followed by chemotherapy *vs* other agent-based chemoradiotherapy, followed by chemotherapy	GITSG (1985)	Unclear	Adequate	Not performed	Adequate
	Li *et al* (2003)	Unclear	Unclear	Not performed	Adequate
	Wilkowski *et al* (2006)	Unclear	Unclear	Not performed	Unclear
Radiotherapy *vs* chemoradiotherapy	Cohen *et al* (2005)	Adequate	Adequate	Not performed	Adequate
	Moertel *et al* (1969)	Unclear	Unclear	Adequate	Adequate
Chemotherapy *vs* chemoradiotherapy, followed by chemotherapy	GITSG (1988)	Adequate	Adequate	Not performed	Adequate
	Klassen *et al* (1985)	Adequate	Unclear	Not performed	Adequate
	Hazel *et al* (1981)	Unclear	Unclear	Not performed	Adequate
	Chauffert *et al* (2006)	Unclear	Unclear	Not performed	Unclear

BSC=best supportive care; FU=fluorouracil.

**Table 2:** Study included in comparison of chemoradiation, followed by chemotherapy *vs* BSC

**Trial**	**Group**	**Mean age and gender**	**Chemotherapy/radio-therapy used and dose**
Shinchi *et al* (2002)	Chemoradiation, followed by chemotherapy (*n*=16)	62.9 years; 36% women, 64% men	50.4 Gy per 28 fractions and continuous-infusion 5FU 200 mg m^−2^ day^−1^
	BSC (*n*=15)	64.6 years; 67% women, 33% men	—

**Table 3:** Study included in comparison of chemoradiation, followed by chemotherapy *vs* radiation

**Trial**	**Group**	**Mean age and gender**	**Chemotherapy/radio-therapy used and dose**
Moertel *et al* (1981)	Chemoradiation, followed by chemotherapy (*n*=31)	62.9 years; 36% women, 64% men	6000 rad, given as 2000 rad over 2 weeks and separated by a 2 weeks' rest period. A total of 5FU – 500 mg m^−2^ day^−1^ on days 1–3 of each 2000 rad radiotherapy course
	Radiation alone (*n*=25)	64.6 years; 67% women, 33% men	6000 rad

**Table 4:** Study included in comparison of 5FU-based chemoradiation, followed by chemotherapy *vs* another chemotherapy agent-based chemoradiation, followed by chemotherapy

**Authors**	**Group (number randomised)**	**Median age and gender**	**Chemotherapy/radio-therapy used and dose**
Wilkowski *et al* (2006)	5FU chemoradiotherapy (*n*=32)	NA	5FU 350 mg m^−2^ irradiation day^−1^+50 Gy conventional radiation
	Gemcitabine+cisplatin chemoradiotherapy (*n*=33)	NA	Gemcitabine 300 mg m^−2^ day^−1^ 30 min infusion, cisplatin 30 mg m^−2^ day^−1^ 60 min infusion on days 1, 8, 22 and 29+50 Gy conventional radiation
Li *et al* (2003)	5FU chemoradiotherapy (*n*=16)	69 years; 12 men, 4 women	500 mgm^−2^ day^−1^ for 3 days, repeated every 2 weeks for 6 weeks+3D conformal radiotherapy 50.4–61.2 Gy
	Gemcitabine chemoradiotherapy (*n*=18)	68.5 years; 13 men, 5 women	600 mg m^−2^ week^−1^ for 6 weeks+3D conformal radiotherapy 50.4–61.2 Gy
GITSG (1985)	5FU chemoradiotherapy (*n*=79)		5FU 500 mg m^−2^ on first 3 days of each radiotherapy course+6000 rad double split course, followed by weekly maintenance with 5FU 500 mg m^−2^ till progression
	Adriamycin chemoradiotherapy (*n*=78)		Adriamycin 15 mg m^−2^ on day 1; thereafter 10 mg m^−2^ week^−1^, for a minimum of five doses+4000 rad continuous course, followed by weekly maintenance with 5FU 500 mg m^−2^ till progression

FU=fluorouracil.

**Table 5:** Studies included in comparison of radiotherapy *vs* chemoradiotherapy

**Trial**	**Group**	**Median age and gender**	**Chemotherapy/radiotherapy used and dose**
Moertel *et al* (1969)	Radiotherapy (*n*=32)	NA	3500–4000 rad by cobalt 60 teletherapy unit
	Chemoradiotherapy (*n*=32)	NA	3500–4000 rad by cobalt 60 teletherapy unit, 5FU 45 mg kg^−1^ on first 3 days of radiotherapy
Cohen *et al* (2005)	Radiotherapy (*n*=49)	62 years; 55 men, 45 women	Radiotherapy 59.4 Gy
	Chemoradiotherapy (*n*=55)	64 years; 67 men, 33 women	Radiotherapy 59.4 Gy, 5FU 1000 mg m^−2^ day^−1^ on days 2–5 and 28–31 of radiotherapy MMC 10 mg m^−2^ on day 2

MMC=mitomycin; 5FU=5-fluorouracil.

**Table 6:** Included studies – chemotherapy *vs* chemoradiotherapy, followed by chemotherapy

**Authors**	**Group (number randomised)**	**Median age and gender**	**Chemotherapy/radiotherapy used and dose**
Hazel *et al* (1981)	Chemo (*n*=15)	NA	5FU 500 mg m^−2^ weekly, methyl CCNU 100 mg m^−2^ every 6 weeks
	Combin rx (*n*=15)	NA	5FU 500 mg m^−2^ weekly, radiotherapy 4600 rad in 4.5 weeks. After completion of chemoradiation, methyl CCNU added
Klassen *et al* (1985)	chemo (*n*=44)	NA; 31 men, 13 women	5FU 600 mg m^−2^ weekly
	Combin rx (*n*=47)	NA; 22 men, 25 women	5FU 600 mg m^−2^ on first days of radiotherapy 4000 rad radiotherapy over 4 weeks After completion of chemoradiation, 5FU 600 mg m^−2^ weekly
GITSG (1988)	Chemo (*n*=21)	60 years; 13 men, 8 women	5FU 600 mg m^−2^ on days 1, 8, 29, 36, streptozocin 1 g m^−2^ every 8 weeks, mitomycin 10 mg m^−2^ on day 1 every 8 weeks
	Combin rx (*n*=22)	61 years; 14 men, 8 women	Radiotherapy 5400 rad over 6 weeks with 5FU 350 mg m^−2^ on first 3 days and last 3 days of radiotherapy. After completion of chemoradiation, chemo-SMF regimen: 5FU 600 mg m^−2^, streptozocin 1 g m^−2^ on days 1, 8, 29, 36 every 8 weeks, mitomycin 5 mg m^−2^ at first dose, then 10 mg m^−2^ every 8 weeks
Chauffert *et al* (2006)	Chemotherapy (*n*=60)	Mean age=60.1 years	Gemcitabine 1000 mg m^−2^ 7q8 weeks initially, then 3q4 weeks
	Combination rx (*n*=59)	Mean age=62.7 years	60 Gy in 6 weeks, with 5FU 300 mg m^−2^ 24 h^−1^ on days 1–5 every week and cisplatin 20 mg m^−2^ day^−1^ on days 1–5 at week 1 and 5. After completion of chemoradiation, gemcitabine 1000 mg m^−2^ 3q4 weeks

C, CCNU=lomustine; chemo=chemotherapy; Combin rx=combination therapy (chemoradiotherapy, followed by chemotherapy); MMC=mitomycin; NA=data not available.
